# Editorial: Recent advances in pediatric inflammatory diseases

**DOI:** 10.3389/fped.2023.1237625

**Published:** 2023-07-27

**Authors:** Begüm Kocatürk, Füsun Özmen, Moshe Arditi, Seza Özen

**Affiliations:** ^1^ Department of Basic Oncology, Hacettepe University Cancer Institute, Ankara, Turkey; ^2^ Department of Pediatrics, Cedars-Sinai Medical Center, Los Angeles, CA, United States; ^3^ Division of Rheumatology, Department of Internal Medicine, Hacettepe University Faculty of Medicine, Ankara, Turkey

**Keywords:** immune response, pediatrics, inflammation, autoimmunity, immune-mediated diseases

**Editorial on the Research Topic** Recent Advances in Pediatric Inflammatory Diseases

The immune system defends the human body from outside invaders and is critical for sustaining life in a hostile environment. It is relatively immature at birth and during infancy, evolves in childhood through exposure to multiple foreign challenges into young and mature adulthood, and gradually declines in old age (1, 2). While impaired immunity may lead to severe infections in infants and children, the immune responses may become exaggerated, leading to inflammatory and autoinflammatory diseases, or may turn against the host, leading to hyper-inflammatory disorders. Understanding the novel molecular mechanisms used by these diseases is necessary to inform the development of more effective treatment strategies.

In the Research Topic “Recent Advances in Pediatric Inflammatory Diseases”, our goal is to share the recent progress on the molecular mechanisms and a current overview on some pediatric inflammatory diseases. A potpourri of pediatric diseases such as Kawasaki disease (KD), multisystem inflammatory syndrome in children (MIS-C), juvenile idiopathic arthritis (JIA)-associated uveitis, and a review of spondyloarthritides (SpA) is presented.

## The protective role of breastfeeding in KD

KD is defined as a systemic vasculitis predominantly affecting small- and medium-sized vessels. At present, KD is the leading cause of acquired heart disease in children in developed countries (3). The etiology of the disease is still unknown; it is suspected to be caused by an abnormal immune response against an infectious agent. During the acute phase of KD, innate immune hyperactivation accompanying an increase in macrophage and neutrophil counts, elevated levels of antigen–antibody complexes, IL-1β, IL-8, IL-6, and IL-17A cytokines, and a Th17-associated immune response are observed (4, 5). The treatment consists of intravenous immunoglobulin (IVIG) together with aspirin; however, the presence of IVIG-resistant patients compromises the success of this therapy (6); thus, preventive measures and alternative treatment strategies need to be explored thoroughly.

Human breast milk has an immunomodulatory role and lowers the risk of developing many immune system-mediated diseases (7). Na et al. use a large cohort to investigate whether breastfeeding also has a protective role against KD development in infants. This study reveals that it indeed reduces the incidence rate of KD for a specific amount of time, and exclusive breastfeeding extends this period even further compared to partial breastfeeding, thus suggesting a prophylactic role of human breast milk on KD vasculitis. The transfer of breast milk-derived immune components such as immunoglobulins and lymphocytes from mother to neonate helps to maintain, modulate, and instruct the infant's immunity. In addition, the organization of neonatal intestinal microbiota via nursing might very well explain the observed effects. The World Health Organization (WHO) recommends exclusive breastfeeding and partial breastfeeding for no less than 6 months and until the age of 2 years, respectively (8, 9), and the study by Na et al. once again underlines its importance.

## The history of MIS-C waves

In 2020, the WHO declared the COVID-19 outbreak a global pandemic (10). In the same year, reports emerged stating the admission of children to hospitals with MIS-C. Intriguingly, the disease progression was found to resemble Kawasaki disease and toxic shock syndrome, whereafter the WHO, RCPCH, and CDC released criteria helping its differential diagnosis. The etiology remains enigmatic, but it is still believed to be an overreaction of the body's immune system possibly triggered by SARS-CoV-2. Sustained monocyte activation, IFN-*γ* signaling induction (11), Vβ21.3+ T-cell expansion (12), and genetic deficits in OAS-RNase L (13) are observed in patients. Kechiche et al. analyze the changing diagnostic course, symptoms, and treatment strategies for MIS-C over time. The decrease in hospitalization duration and mean diagnosis time indicates the advancement in the disease management. In addition, changes in the treatment strategy, primarily comprising the increased use of corticosteroids, seem to help to decrease the disease severity.

## A summary of the current knowledge on SpA

SpA cover a group of chronic inflammatory diseases affecting the axial and peripheral joints, skin, eye, and gut (14). The etiology of SpA is also not known; thus, the diagnosis criteria and therapy regimens need to be routinely revised. Kocatürk et al. present a detailed overview of the current knowledge, encompassing the classification criteria, treatment options, and plausible risk factors associated with ankylosing spondylitis and enthesitis-related arthritis in particular. This review article also provides comprehensive evidence for HLA-B27-driven immune activation in disease pathogenesis in addition to antigen presentation, protein degradation, and microbiome-directed processes. The side effects of and inadequate response to the current treatments raise unfolded protein response and IL-23/IL-17 as new therapy targets. However, further research is warranted.

## Deciphering novel autoantigens for JIA-associated uveitis

JIA is defined as an arthritis with an onset younger than 16 years of age that persists for at least 6 weeks, and it remains the most common cause of chronic arthritis in childhood (15). Diagnosis of the disease is based on clinical evaluation; however, the lack of a reliable biomarker complicates this process. A variety of autoantibodies such as anti-nuclear antibodies (ANA), rheumatoid factor, and anti-citrullinated protein antibodies show association with JIA, whereas this association is not strong for all subtypes (16). Their presence does not explicitly confirm JIA diagnosis but serves to rule out other conditions or pinpoint extra risk factors. For instance, ANA positivity is an indicator for increased risk of uveitis, and patients are advised to have regular eye exams as it may result in permanent vision loss (17).

The critical role of autoantibodies in uveitis progression is underlined by studies showing prominent B and plasma cell infiltrate in ocular inflammatory infiltrate (18) and is further supported by the success of B-cell depletion therapy in JIA-associated uveitis treatment (19). As ANA positivity is not specific to JIA-associated uveitis, Arve-Butler et al. identify novel autoantigens. They use two delicate methodologies in their discoveries, namely, a peptide array and an immunoprecipitation-based technique subsequently complemented with a bead array. Their analysis reveals 17 autoantigens showing differential reactivity between patients with and without uveitis. Another interesting aspect of autoantigens is their post-translational modification (PTM) profile. Autoantibodies directed against citrullinated or carbamylated proteins are detected in patients with JIA (16), and proteins carrying these PTMs are known to have arthritogenic properties (20, 21). Among the autoantigens identified by Arve-Butler et al., the citrullination of enolase has been previously described in JIA (22); therefore, developing an understanding of the PTM profile of the other targets deserves our attention.

## Concluding remarks

Many of the established and newly emerging pediatric inflammatory diseases still preserve their mysterious origin. Each day, with the advancement of medicine, we get one step closer to better understanding these diseases. The identification of preventive measures, advancement in treatment strategies, and determination of risk factors help to unearth the molecular basis of these disorders, thus supporting and improving the identification of targeted therapies. We hope that the articles of this research topic serve as further encouragement to researchers engaged in finding the missing pieces.
